# The Hippo signaling pathway in gastric cancer: Targeting Hippo for GC treatment

**DOI:** 10.3724/abbs.2023038

**Published:** 2023-03-16

**Authors:** Zhifa Cao, Liwei An, Yi Han, Shi Jiao, Zhaocai Zhou

**Affiliations:** 1 Department of Stomatology Shanghai Tenth People’s Hospital Department of Biochemistry and Molecular Biology Tongji University School of Medicine Shanghai 200072 China; 2 CAS Center for Excellence in Molecular Cell Science Institute of Biochemistry and Cell Biology Shanghai Institutes for Biological Sciences Chinese Academy of Sciences University of Chinese Academy of Sciences Shanghai 200031 China; 3 State Key Laboratory of Genetic Engineering School of Life Sciences Zhongshan Hospital Fudan University Shanghai 200438 China; 4 Collaborative Innovation Center for Cancer Personalized Medicine School of Public Health Nanjing Medical University Nanjing 211166 China

**Keywords:** gastric cancer, Hippo pathway, YAP/TAZ, mechanism, targeted therapy

## Abstract

Gastric cancer (GC) is an aggressive malignant disease which still lacks effective early diagnosis markers and targeted therapies, representing the fourth-leading cause of cancer-associated death worldwide. The Hippo signaling pathway plays crucial roles in organ size control and tissue homeostasis under physiological conditions, yet its aberrations have been closely associated with several hallmarks of cancer. The last decade witnessed a burst of investigations dissecting how Hippo dysregulation contributes to tumorigenesis, highlighting the therapeutic potential of targeting this pathway for tumor intervention. In this review, we systemically document studies on the Hippo pathway in the contexts of gastric tumor initiation, progression, metastasis, acquired drug resistance, and the emerging development of Hippo-targeting strategies. By summarizing major open questions in this field, we aim to inspire further in-depth understanding of Hippo signaling in GC development, as well as the translational implications of targeting Hippo for GC treatment.

## Introduction

Gastric cancer (GC) remains one of the most malignant tumor types worldwide, ranking as the fifth- and fourth-leading causes of global morbidity and mortality, respectively [1]. Although gradually declining in most parts of the world during the past 20 years, the occurrence of GC is still growing in high-risk regions such as the Eastern Asian countries [2]. For example, the number of newly diagnosed GC cases increased in China from 400,000 in 2015 to 480,000 in 2020, and the accompanying deaths dramatically increased from 290,000 to 380,000, both of which accounted for nearly 50% of all newly diagnosed cases and deaths worldwide [3]. GC is a complex disease that is affected by multiple genetic, epigenetic and environmental factors, including *Helicobacter pylori* ( *H*. *pylori*) infection, EBV infection, age, high salt intake, and iron deficiency [4]. Due to the prevalence of modern lifestyles such as smoking, alcohol, unbalanced diet, and obesity, GC incidence is rapidly growing in the young population (age below 45 years old) [5].

Traditionally, GC is mainly evaluated via histological phenotypes such as the Borrmann, Lauren, and WHO classifications [6]. Among them, the Lauren classification is widely used in the clinic to divide GC into intestinal and diffuse types [6]. In 2014, two landmark studies led by The Cancer Genome Atlas (TCGA) and Asian Cancer Research Group (ACRG) independently proposed molecular subtype classifications of GC with partially overlapping and complementary features [ 7, 8] . Briefly, the TCGA group defined GC into four subtypes: Epstein-Barr virus-positive subtype (EBV ^+^, 9%), microsatellite unstable (MSI, 22%), chromosomal instability (CIN, 50%), and genomic stable (GS, 20%) [7]. In addition to molecular alterations, the ACRG group further combined analysis of corresponding clinical outcomes and thus divided GC into MSI, microsatellite stable with epithelial-mesenchymal transition (MSS/EMT), MSS positive for TP53-active (MSS/TP53 ^+^), and MSS with loss of TP53 (MSS/TP53 ^–^) [8]. Among them, the MSS/EMT subtype shows the worst prognosis and tends to occur at an earlier stage, whereas the MSS/TP53 ^+^ subtype positively correlates with EBV infection [8]. Overall, these profiles of genetic alterations in GC subtypes provide a basis to investigate the underlying molecular mechanisms driving gastric tumorigenesis.

Signaling pathways such as the cell cycle, Hippo, Myc, Notch, TGFβ, p53 and Wnt/β-catenin pathways have been widely implicated in tumor initiation and progression [ 9, 10] . Notably, some tumor-specific alterations of these signaling pathways also create vulnerabilities that may be used to develop targeted therapies [11]. Indeed, among the targeted drugs approved by the FDA for GC treatment (Trastuzumab, fam-trastuzumab, pembrolizumab, disitamab, ramucirumab, Keytruda and Opdovo), most target dysregulated signal transduction factors such as epidermal growth factor receptor 2 (HER2) and vascular endothelial growth factor receptor (VEGFR) [12]. Likewise, Apatinib, the only chemical drug approved in China, also targets VEGFR for GC treatment [13]. Taken together, these successful targeting paradigms attract increasing attention to characterize the relevant alterations of key signaling pathways between normal and cancer cells and how these dysregulated signals contribute to GC tumorigenesis, hoping to identify novel therapeutic targets [14].

**Figure 1:**
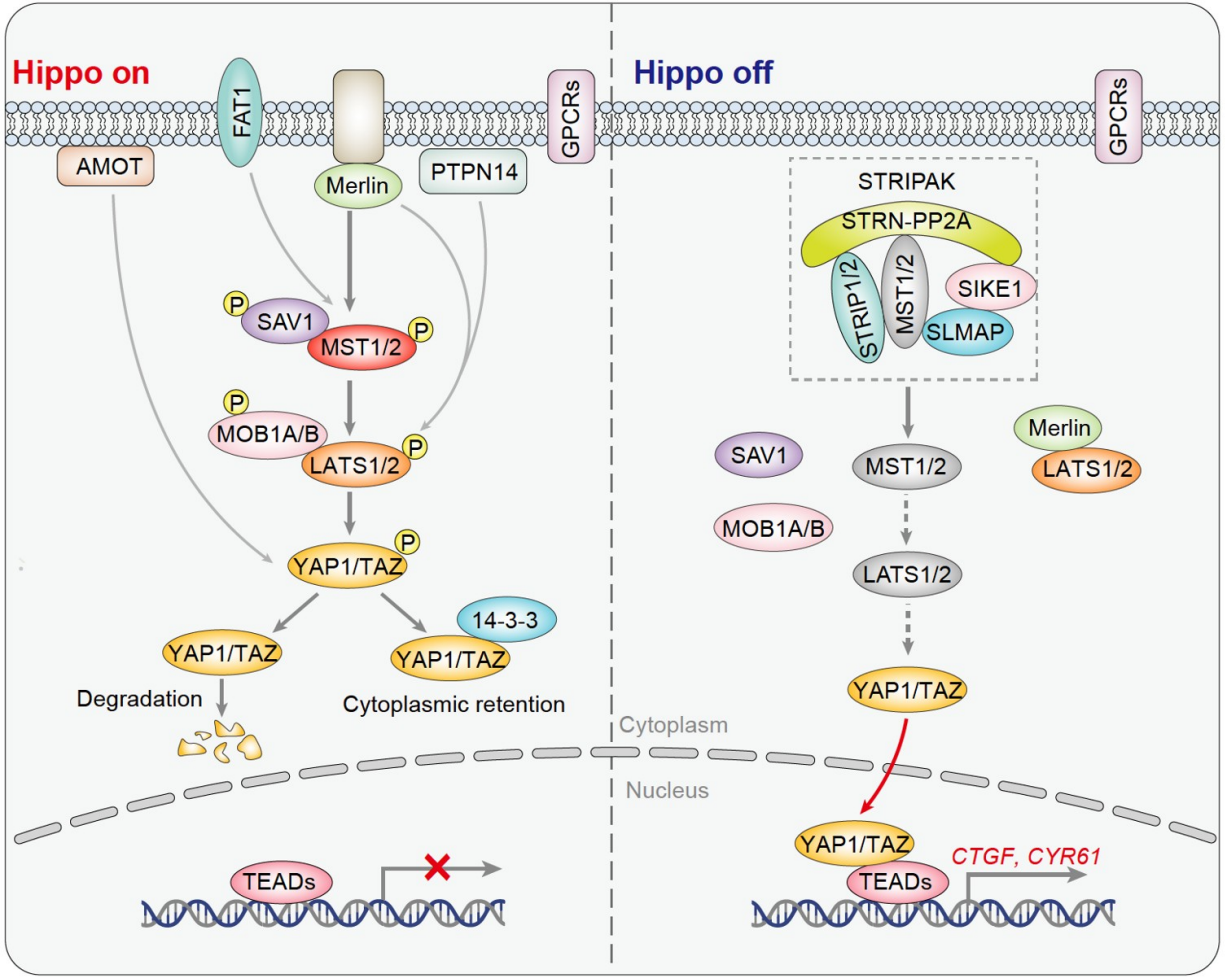
Figure 1 A schematic illustration of the Hippo-YAP signaling pathway In response to different extracellular stimuli, the activity of MST1/2 is regulated in a phosphorylation-dependent manner by upstream factors such as AMOT, FAT1, Merlin and PTPN14. As part of the kinase cascade, MST1/2 can activate LATS1/2-mediated YAP/TAZ inactivation. Phosphorylated YAP/TAZ will stay in the cytoplasm and undergo proteasome degradation (Hippo on, left panel). Meanwhile, factors such as STRIPAK and GPCRs can trigger a dephosphorylation cascade of MST1/2-LATS1/2-YAP/TAZ, which eventually induces YAP/TAZ nuclear translocation, where they bind to the TEAD family of transcription factors to regulate downstream target gene expression (Hippo off, right panel).

The Hippo pathway is an evolutionarily conserved phosphorylation-dependent signaling cascade, which plays crucial roles in organ size control, tissue homeostasis and immune modulation [ 15, 16] . Briefly, in the well-characterized Hippo signaling cascade (Hippo on), the mammalian Ste20-like kinases MST1/2 (the Hippo kinases), aided by the adapter factor Salvador homolog 1 (SAV1), first phosphorylate the large tumor suppressor 1/2 kinases (LATS1/2), which in turn phosphorylate the downstream transcriptional coactivator yes-associated protein 1 (YAP) or the WW domain-containing transcription regulator 1 (TAZ) ( Figure 1). Phosphorylated YAP/TAZ will then be sequestered in the cytoplasm via interaction with the 14-3-3 complex and eventually undergo ubiquitination-dependent degradation ( Figure 1). In contrast, once phosphorylation-dependent signaling is shut down (Hippo off), YAP/TAZ can enter the nucleus to bind with the TEA domain family of transcription factors (TEAD1-4), forming a complex to regulate downstream target gene expression that promotes cell proliferation and inhibits apoptosis ( Figure 1). An important feature of the Hippo pathway is its response to environmental cues such as tight junctions, nutrition, GPCR activation, and mechanical stress [17]. Correspondingly, a number of positive regulators, such as FAT atypical cadherin 1 (FAT1) [ 18, 19] , AMOT [20], and PTPN14 [ 21, 22] , and negative regulators, such as striatin-interacting phosphatases and kinases (STRIPAK) [ 23– 25] and G-protein-coupled receptors (GPCRs) [26], have been identified in the upstream of the Hippo pathway to orchestrate environmental cues into signals governing gene transcription [27] ( Figure 1).

Accumulating evidence suggests that Hippo pathway dysregulation and aberrant YAP/TAZ-TEAD activation are associated with various diseases, most notably cancer, making this pathway an attractive target for therapeutic intervention [28]. Of note, ever since the observations that upregulation of YAP exhibits oncogenic properties and its nuclear accumulation predicts poor prognosis of GC [ 29, 30] , great progress has been made in the last decade in understanding the pathological roles of YAP activation in GC development. Thus, we reviewed the current knowledge related to alterations in the Hippo signaling pathway in response to GC risks, as well as how dysregulated Hippo signaling contributes to GC tumorigenesis, aiming to facilitate the development of translational Hippo-targeting strategies for GC diagnosis and personalized medicine.

## The Hippo Pathway in GC

The Hippo pathway is frequently dysregulated in a wide range of human malignancies. For example, aberrant YAP/TAZ transactivation, often associated with their elevated expression, enhanced nuclear localization, and amplification of downstream target genes, is thought to play multiple roles in tumor initiation, progression, metastasis and acquired drug resistance in GC [ 31, 32] . In addition to hyperactivation of YAP/TAZ [ 29, 30] , TEADs [ 33, 34] have also been found to be frequently overexpressed in GC. In contrast to its classical role in the Hippo pathway, MST2 kinase was recently shown to play an oncogenic role in GC [35]. Interestingly, disease-related mutations were rarely found in the Hippo core components, raising the question of how exactly this signaling pathway regulates the initiation and progression of GC [36].

### Hippo signaling in GC initiation

Considering the wide range of genetic and environmental factors involved in GC pathology, one key question awaiting investigation is how specific types of GC are initiated under different contexts. Among the multiple exogenous GC risk factors, *H*. *pylori* infection is the leading cause and has been recognized as a class I carcinogen by the WHO since 1994 [37]. In most cases, *H*. *pylori* infection leads to chronic inflammation of the gastric mucosa, which can slowly evolve into atrophy, metaplasia, dysplasia, and gastric carcinoma, often in a period of several decades [ 38, 39] . Mechanistically, *H*. *pylori* infection dysregulates the signaling transduction networks in gastric mucosal epithelia via both outer membrane proteins and multiple virulence factors injected into the host cells [40]. For example, cytotoxin-associated gene A (CagA), the best known virulence factor associated with GC initiation and development, has been shown to regulate multiple signaling pathways, such as Wnt/β-Catenin [41], AMPK [41], PI3-kinase/Akt [42], JAK/STAT [43], and ERK [ 44, 45] , leading to cell proliferation, inflammatory cell infiltration, and overall survival.

**Figure 2:**
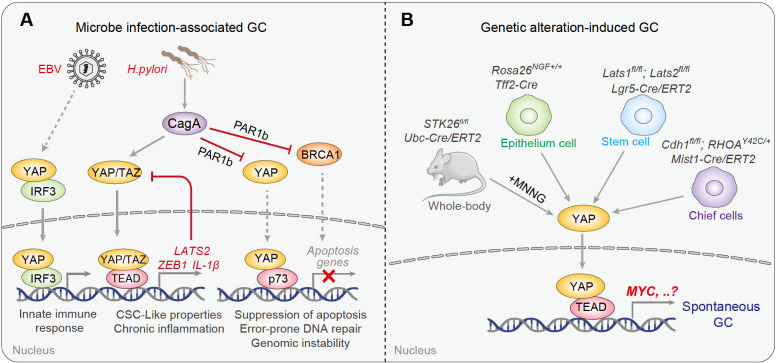
Figure 2 Summary of Hippo signaling in GC initiation (A) Current knowledge related to Hippo signaling in response to H. pylori and EBV infection-associated GC initiation. (B) Summary of YAP-related spontaneous GC initiation in genetically engineered mouse models. The key oncogenic downstream target genes remain elusive except MYC.

In the last five years, several groups have independently investigated the roles of Hippo signaling in response to *H*. *pylori* infection-associated gastric tumorigenesis ( Figure 2A) [ 46– 52] . Upon *H*. *pylori* infection, both YAP [ 46, 48, 49] and TAZ [47] were translocated into the nucleus, and YAP/TAZ remained highly activated in human gastric carcinoma, suggesting active involvement of the Hippo pathway during *H*. *pylori-*induced early GC initiation. Functionally, YAP/TAZ signaling can promote epithelial–mesenchymal transition (EMT) and cancer stem cell-like properties by activating the expressions of related target genes [ 46, 47, 49] . Meanwhile, *H*. *pylori* infection may also elevate the expression of LATS2 kinase at the late stage, which counteracts the role of YAP during the processes of EMT and intestinal metaplasia, indicating a biphasic pattern of Hippo in response to *H*. *pylori* infection ( Figure 2A) [49].

A recent *in vivo* study revealed sequential activation of Wnt and YAP signaling essential for the development of chronic *H*. *pylori* infection-induced premalignant gastric metaplasia [51]. Moreover, activated YAP-TEAD can also drive the transcription of inflammatory cytokine factors such as IL-1β upon *H*. *pylori* infection, accounting for the establishment of a chronic inflammatory microenvironment [48]. Interestingly, ectopic expression of CagA, by forming a complex with the polarity-regulating kinase PAR1b, can suppress nuclear translocation of both BRCA1 and YAP to initiate gastric carcinogenesis with a BRCAness-associated genome instability phenotype [50]. In this context, activation of Hippo signaling circumvents YAP/p73-mediated apoptosis of DNA-damaged cells, giving these cells time to repair DSBs through the error-prone repair pathway [50] ( Figure 2A).

It is believed that the EBV ^+^ GC subtype is sensitive to PD-1/PD-L1 blockade treatment, indicating that EBV infection represents a favorable biomarker for immunotherapy [53]. TCGA revealed amplification of the *PD-L1* genomic locus in the EBV ^+^ GC subtype, yet the underlying mechanism is still poorly understood [7]. In fact, it remains unclear how EBV contributes to GC initiation and helps to reshape the immune microenvironment during GC progression. In this regard, EBV infection may activate the innate immune response via IRF3, which in turn forms a complex with YAP to cotranslocate into the nucleus, eventually resulting in both YAP- and IRF3-dependent gene transcription [52] ( Figure 2A). These findings suggest that the Hippo pathway is involved in microbe-mediated GC initiation. In the future, it would be highly interesting to explore the potential role and mechanism of Hippo in GC pathology from the perspective of bacteria other than *H*. *pylori* and their interactions with other components within the tumor ecosystem.

In addition to environmental risk factors, whether Hippo-YAP signaling is involved in genetically induced GC initiation represents another important and puzzling issue. For example, is YAP/TAZ activation a cause or consequence of GC? In this regard, a research group from South Korea first explored the roles of YAP/TAZ activation in driving GC tumorigenesis using genetic mouse models [54]. To this end, they specifically deleted LATS1/2 in *Lgr5 ^+^* stem cells ( *Lats1* ^*fl*/ *fl*^; *Lats2* ^*fl*/ *fl*^; *Lgr5-CreERT*) to enable cell-type specific YAP/TAZ activation. They found a hyperplasia phenotype 8 weeks after *Lats1*/ *2* knockout, which eventually develops into intramucosal invasive carcinoma at 20-24 weeks, indicating that YAP/TAZ activation is a direct driver of GC initiation [54]. In this process, *MYC* could be a direct transcriptional target responsible for YAP-induced tumorigenesis, blockade of which could efficiently rescue such tumor initiation ( Figure 2B) [54]. Meanwhile, Timothy Wang’s group found that mice with specific knock-in of nerve growth factor (NGF) in Tff2 ^+^ cells ( *Tff2-Cre*; *Rosa26* ^*NGF*^^+/+^) also spontaneously developed metaplasia and dysplasia in the stomach at 8 months, which eventually developed into large gastric tumors with intramucosal adenocarcinoma at 18 months [55]. Further molecular delineation revealed YAP activation under this condition ( Figure 2B) [55]. Recently, MST4 was identified as a noncanonical Hippo kinase, deficiency of which positively correlated with YAP activation and worse GC prognosis [56]. Importantly, whole-body depletion of MST4 ( *Ubc-Cre*/ *ERT2*; *STK26* ^*fl*/ *fl*^) dramatically accelerated MNNG-induced GC tumorigenesis, again suggesting that activated YAP contributes to GC initiation and development ( Figure 2B) [56].

At this stage, further studies are still required to clarify a direct role of Hippo signaling in early GC development, such as possible off-target effects caused by genetic manipulation [ 54– 56] . Meanwhile, genome-wide molecular profiling of GC has identified a set of genetic alterations in tumor suppressors such as *TP53*, *CDH1*, *RHOA*, *ARID1A*, and *RNF43* [ 7, 8] . Whether and how Hippo signaling is involved in these genetic alteration-associated GC initiation warrants further investigation. In this regard, a recent study utilized genetically engineered mouse models ( *Cdh1* ^*fl*/ *fl*^; *Rhoa* ^*Y42C*^^/+^; *Mist1-Cre*/ *ERT2*) to recapitulate recurrent *CDH1* loss coupled with *RHOA ^Y42C^* genomic alterations, which are frequently observed in genomically stable subtype GC [57]. Both activated YAP/TAZ and β-catenin are required for transformation and diffuse gastric cancer development ( Figure 2B) [57].

### Hippo signaling in GC progression

**Figure 3:**
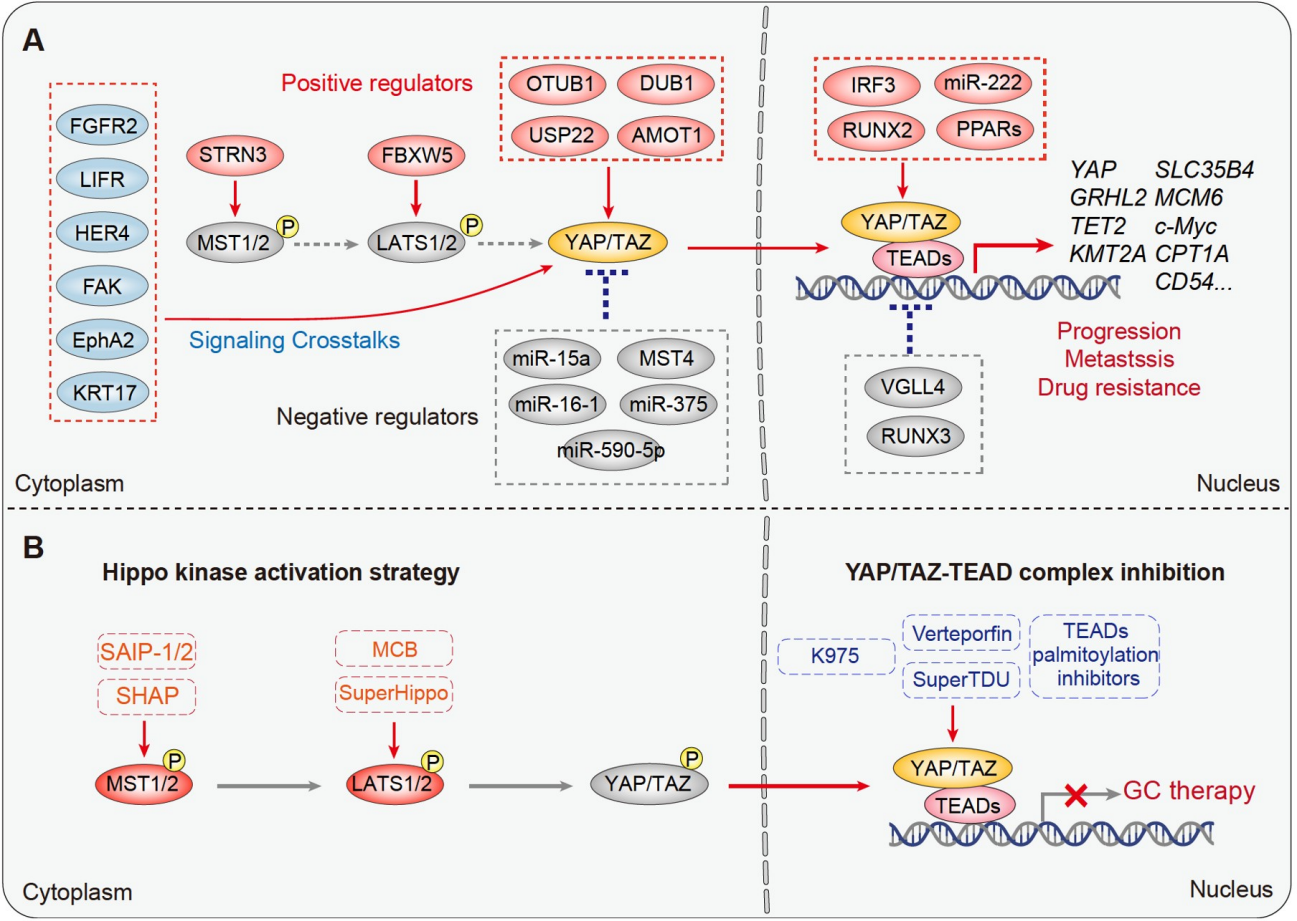
Figure 3 Dysregulation of Hippo signaling in GC and targeting strategies (A) Current knowledge related to how YAP/TAZ is hyperactivated in GC, including loss of negative regulators, upregulation of positive regulators, and assembly of tight YAP/TAZ-TEAD transcriptional complexes. The possible downstream effectors responsible for GC progression, metastasis, and drug resistance were also demonstrated. (B) Summary of Hippo-targeting strategies for cancer therapy. In the cytoplasm, peptides or chemical agonists were developed to restore the tumor-suppressing activity of the Hippo kinases (Hippo kinase activation strategy). Alternatively, the YAP-TEAD complex can also be targeted to suppress transcriptional activity (YAP-TEAD complex inhibition).

Relative to its role in GC initiation, Hippo signaling has been extensively implicated in GC progression, including proliferation, immune escape, and tumor innervation [58]. In general, three major types of dysregulations have been documented for Hippo signaling during YAP-driven GC development: reduction or loss of inhibitory negative signals, upregulation of YAP/TAZ expression, and formation of enhanced YAP/TAZ-TEAD transcriptional complexes ( Figure 3A). Regardless of the specific type of dysregulation, a wide range of downstream effectors can be activated to promote tumor progression in diverse contexts of the tumor microenvironment (TME).

Multiple negative regulators of YAP identified under physiological conditions, such as cytoplasmic MST1/2 [59], LATS1 [60], and MST4 [56], as well as nuclear factors VGLL4 [61] and RUNX3 [62], were found to be dramatically reduced or inactivated during GC development, resulting in YAP-dependent GC tumor growth. For example, as the core component of the STRIPAK complex, the expression of STRN3 was dramatically increased in GC, causing dephosphorylation of MST1/2 and subsequent hyperactivation of YAP [59]. Similarly, the E3 ubiquitin ligase FBXW5, which is upregulated in GC, can promote LATS1 ubiquitination and degradation, therefore indirectly causing YAP activation and subsequent GC proliferation and invasion [60]. As a noncanonical member of the mammalian Hippo family of kinases, MST4 works in parallel with the MST1/2-LATS1/2 axis to limit YAP activity via phosphorylation of the Thr83 residues in response to serum starvation; this MST4-YAP signaling was greatly attenuated during GC development [56]. Normally, VGLL4 can restrain YAP-TEAD transcriptional activity by competing with YAP for binding to TEADs, yet VGLL4 expression was found to be gradually reduced during GC development [61]. Moreover, the oncogenic microRNA miR-222 may target VGLL4 for degradation, thus resulting in hyperactivation of YAP [63]. Likewise, the transcription factor RUNX3 has been reported to compete with TEAD4 for complexing with YAP [62]. In this regard, loss-of-function mutants of RUNX3 identified in GC could facilitate YAP-TEAD4 complex formation and thus promote GC progression [64]. In addition, miR-15a, miR-16-1 [65], miR-375 [66], and miR-590-5p [ 67, 68] can similarly act as tumor suppressors by targeting YAP degradation; however, downregulation of these microRNAs may contribute to YAP-dependent GC growth ( Figure 3A).

Alternatively, cancer cells can activate YAP/TAZ signaling by direct modulation of their expression. For example, several cytoplasmic factors, including the deubiquitinating enzymes OTUB1 [69], USP22 [70], AMOT1 [71], and DUB1 [72], have been reported to protect the YAP [ 69– 71] or TAZ [72] protein from proteasome-mediated degradation. These positive regulators are dramatically elevated in GC samples, correlate with YAP/TAZ nuclear localization, and predict unfavorable prognosis [ 69, 71, 72] . In addition to protein stabilization, the transcription of *YAP* was also elevated in GC. For example, YAP was found to be a direct target of the transcription factor RUNX2, whose overexpression promotes YAP-dependent GC progression [73]. Furthermore, a recent study reported a positive feedback loop between epigenetic regulation, YAP transcription and activation in response to matrix stiffness [74]. They found that the methylation status of the *YAP* promoter region is gradually reduced with increased extracellular matrix stiffness, leading to enhanced YAP transcription and activation in the tumor. Activated YAP signaling further promotes the transcription of methylation-inhibitory genes such as *GRHL2*, *TET2*, and *KMT2A*, which augments the reduction in YAP methylation to eventually promote GC progression [74] ( Figure 3A).

Finally, crosstalk of Hippo-YAP signaling with other pathways and factors has been extensively documented to play important pathological roles in tumorigenesis, including GC. In this regard, both FGF18-FGFR2 [75] and leukemia inhibitory factor (LIF)-LIFR [ 76– 78] signaling have been reported to activate YAP and thus promote gastric carcinogenesis. Additionally, galectin-3 [79], the linc01133-YES1 axis [80] and the long noncoding RNA HCG18 [81] were shown to promote YAP nuclear translocation. Moreover, peroxisome proliferator-activated receptors (PPARs) can interact with and promote YAP-TEAD4 complex loading onto the promoter of *SOX9*, resulting in SOX9-dependent GC progression [82]. Meanwhile, activation of YAP can drive transcription of multiple oncogenic downstream targets, such as solute carrier family 35 member B4 (SLC35B4) [83], c-Myc [79], mini-chromosome maintenance complex component 6 (MCM6) [84], and long noncoding RNAs, such as MNX1-AS1 [85], to promote cancer cell proliferation, migration, and invasion. Genetic depletion or pharmacological inhibition of these downstream effectors could reverse many YAP-dependent GC phenotypes. Recently, a unique set of YAP signature genes, including CD54, was identified in GC-associated neutrophils [86]. Moreover, a YAP-CD54 signaling axis is required for the development and antitumor activity of these neutrophils, the dysregulation of which promotes GC progression [86] ( Figure 3A).

### Hippo signaling in GC metastasis

In general, tumor metastasis accounts for more than 90% of cancer-associated deaths [87]. Studies in lung and breast cancers have implicated YAP/TAZ activation in tumor metastasis [ 88– 90] . Regarding GC, although local lymph node metastasis (LNM) is most frequently observed, advanced GCs predominantly metastasize to the peritoneal region, which acts as a hallmark of incurable treatment and worse prognosis [91]. Recently, two studies independently revealed the oncogenic role of YAP/TAZ in GC peritoneal metastasis (PM) [ 92, 93] . On the one hand, the Song group found that YAP was specifically upregulated in tumoral cells but not in other cells coexisting in the ascites of GC patients [92]. Importantly, RNA-Seq of PM samples confirmed that the expression of YAP is significantly associated with cancer stem cell (CSC) biomarkers such as *SOX9*, *HES1*, *CD133* and *ALDH1A1*, suggesting that these YAP ^high^ tumor cells displayed stemness properties. Indeed, further *in vivo* analysis revealed that such YAP ^high^ tumor cells are more likely to form PDX/PDO tumors, a process that can be attenuated by pharmacological inhibition of YAP activity, implying an essential role of YAP activation in GC peritoneal metastasis [92]. In contrast, the Mano group stratified ascites-disseminated GC into two distinct subtypes, namely, non-EMT and active EMT, via multiomic profiling of tumor cells purified from GC ascites. Interestingly, Hippo pathway activation, illustrated by upregulation of TEAD1/2/4 and TAZ but not YAP, was specifically identified in the active EMT subtype with worse prognosis. Treatment with K975, a small molecular inhibitor of the YAP/TAZ-TEAD interaction, dramatically suppressed EMT-derived GC metastasis and displayed an apparent synergistic effect when in combination with a MEK1/2 inhibitor [93]. Nevertheless, the discrepancy of YAP or TAZ in GC peritoneal metastasis may arise from the heterogeneity of tumor cells in GC ascites. In this regard, a scRNA-Seq study deciphered the cellular landscape of GC ascites, providing a basis to re-examine the cell-type specific function and dysregulation of Hippo in GC peritoneal metastasis [94].

In addition, the Hippo pathway is also implicated in the local LNM of GC patients. For example, activated YAP is involved in the intercellular communication between lymph node metastasis-derived gastric cancer cells (LNM-GC) and infiltrated mesenchymal stem cells (BM-MSCs) via exosomal Wnt5a [95]. Such a reprogrammed metastatic lymph node microenvironment can further promote GC progression [95]. Resembling this model, a recent study revealed that LNM-GC can transmit metastatic capacity to primary GC cells via the exosomal CD44-triggered RhoA-YAP-CPT1A signaling cascade, indicative of multiple regulatory networks of YAP activation in GC lymph node metastasis [96]. In addition, several upstream regulators, such as Netrin-1 [97], SCD1 [98] and CLDN6 [99], have also been shown to promote GC metastasis by regulating YAP-dependent transcriptional activities. At this stage, however, it remains elusive exactly how YAP activation may directly drive GC metastasis. One possibility is to regulate actin dynamics by activating the transcription of GTPase ARHGAP29, whose overexpression has been positively correlated with GC metastasis and shorter survival of GC patients [100]. In diffuse gastric cancer, loss of the intermediate filament KRT17 similarly induces reorganization of the cytoskeleton, which further activates YAP-mediated IL6 expression, contributing to the enhanced metastatic ability of GC cells [101].

### Hippo signaling in GC drug resistance

Adjuvant chemotherapy and surgical resection remain the major treatments for GC in the clinic but face a great challenge of variable clinical outcomes due to the high heterogeneity of GC [102]. Meanwhile, targeted drugs (such as HER2 or VEGF monoclonal antibodies) and immunotherapy (such as PD-1 or PD-L1 blockade) are emerging as first-line treatments for advanced GC [ 31, 103] . Nevertheless, issues of drug resistance or even hyperprogression represent major obstacles for patients to benefit from both chemotherapy and targeted therapy.

Dysregulation of Hippo signaling has also been closely associated with acquired drug resistance in different tumor types, such as promoting the DNA repair capacity in breast cancer [104]. Additionally, several studies have revealed that dysregulated YAP signaling promotes acquired drug resistance in GC. For example, extracellular vesicles (EVs) from cancer-associated fibroblasts (CAFs) can induce tubular network formation and subsequent GC resistance to chemotherapy [105]. Mechanistically, Annexin A6-containing EVs were found to activate β1 integrin-focal adhesion kinase (FAK)-YAP signaling, triggering acquired drug resistance [105]. Similarly, the cell surface erythropoietin-producing hepatocellular receptor A2 (EphA2) has also been reported to induce chemotherapy resistance by increasing YAP stability and its nuclear localization [106] ( Figure 3A). In contrast, the E3 ubiquitin ligase STUB1 can promote YAP protein turnover and result in GC chemosensitivity [107]. Collectively, these studies highlight an essential role for YAP activation in GC acquisition of drug resistance. Moreover, dysregulation of the HER4-YAP axis has been shown to be responsible for trastuzumab resistance in HER2-positive metastatic GC [108] ( Figure 3A). In this case, the HER4-YAP axis appears to activate the PI3K-Akt signaling pathway and suppress apoptosis to induce EMT-mediated trastuzumab resistance [ 108, 109] . In addition to YAP activation, MST1/2 kinases were recently shown to enter the nucleus to directly suppress the DNA repair capacity upon radio- or chemotherapy treatment [110]. Genetic depletion or pharmacological inhibition of MST1/2 dramatically enhanced GC resistance to chemotherapy [110], suggesting a diverse model of action for Hippo to modulate GC drug resistance.

## Targeting the Hippo Pathway in GC

Much attention has been drawn to targeting Hippo-YAP signaling for cancer therapy. Roughly, the current strategies are either by inhibiting the nuclear translocation of YAP in the cytoplasm or by disrupting YAP-TEAD4 complex assembly in the nucleus ( Figure 3B). In 2014, verteporfin (VP) was identified as an inhibitor of YAP that may disrupt the YAP-TEAD4 association, providing the first proof of principle that inhibiting the YAP-TEAD4 interaction is a pharmacologically viable strategy [111]. Indeed, VP has displayed excellent antitumorigenic properties against GC progression *in vivo* [112]. Later, VGLL4 was identified as a natural endogenous antagonist of YAP, and a VGLL4-mimicking peptide named “super-TDU” was thus developed to compete with YAP for TEAD4 binding [61]. As expected, super-TDU efficiently suppressed YAP-driven GC growth both *in vitro* and *in vivo* [61]. Following this scenario, RUNX3 and FAM181A/B were found to similarly disrupt YAP-TEAD complex formation [ 64, 113] . More recently, K975 was also screened out by covalently binding to the YAP-binding domain of TEAD4, acting as a potent YAP-TEAD4 association inhibitor [114]. On the other hand, since palmitoylation is required for TEAD to associate with YAP/TAZ [115], great efforts have been made to search for inhibitors of TEAD palmitoylation [116]. To date, several inhibitors, such as DC-TEADin02 [117], Compound 2 [118], TM2 [119], MGH-CP1 [120], and MYF-03-69 [121], have been reported to either covalently or noncovalently suppress TEAD activity. Considering the ubiquitous TEAD palmitoylation across tumor types as well as the overexpression of TEAD protein in GC, it is likely that these inhibitors may also inhibit GC progression ( Figure 3B).

Apart from targeting YAP-TEAD complex formation in the nucleus, an alternative approach termed the “Hippo kinase activation strategy” is emerging. Several studies have exemplified that enhancing or reactivating upstream kinase activity can promote YAP phosphorylation and therefore inhibit its nuclear translocation. For example, a STRN3-derived Hippo-activating peptide (SHAP) has been developed to selectively disrupt the PP2A-STRN3 interaction and therefore restore Hippo kinase activity [59]. Treatment with SHAP strongly regressed GC growth with the highest sensitivity for poorly differentiated but STRN3-overexpressing tumors [59]. Based on the assembly of the STRIPAK complex and recruitment of MST1/2 [ 25, 122] , additional Hippo-activating peptides were designed, termed STRIPAK assembly inhibitor peptides 1 and 2 (SAIP-1/2) [110]. Treatment with these Hippo-activating peptides (either SHAP or SAIP-1/2) efficiently reactivated MST1/2 kinase activity and suppressed DNA repair capacity, desensitizing GC cells to radiochemotherapy [110]. More strikingly, the attenuated DNA repair capacity induced by Hippo-activating peptides creates an opportunity for synthetic lethality when used in combination with PARP inhibitors, providing a translational strategy of targeted therapy for GC treatment [110]. In a similar case, WWC proteins (WWC1/2/3) were identified as key adaptor factors that directly interact with and activate LATS1/2 kinase activity [123]. Based on this finding, a SuperHippo peptide covering the WWC1/2/3-LATS1/2 interaction interface was thus developed to robustly activate LATS1/2 kinase activity, demonstrating excellent tumor suppression effects in multiple tumor models [123]. In addition, the small molecular natural product microcolin B (MCB) was recently identified to target phosphatidylinositol transfer proteins α and β (PITPα/β), indirectly causing LATS1/2 activation [124]. Similarly, the natural compound usolic acid extracted from herbal plants has also been shown to increase the expression of Hippo kinases and suppress YAP activation and GC progression *in vitro* [125]. Taken together, these studies vigorously validated the concept of “Hippo kinase activation” as a promising strategy for YAP-targeting therapy ( Figure 3B).

## Conclusions and Perspectives

As described above, dysregulation of the Hippo pathway has been closely associated with GC initiation, progression, metastasis, and acquired drug resistance. Despite rapid progress in elucidating the role of Hippo in GC, knowledge gaps still exist before comprehensively deciphering the pathological function of Hippo in GC tumorigenesis.

**Figure 4:**
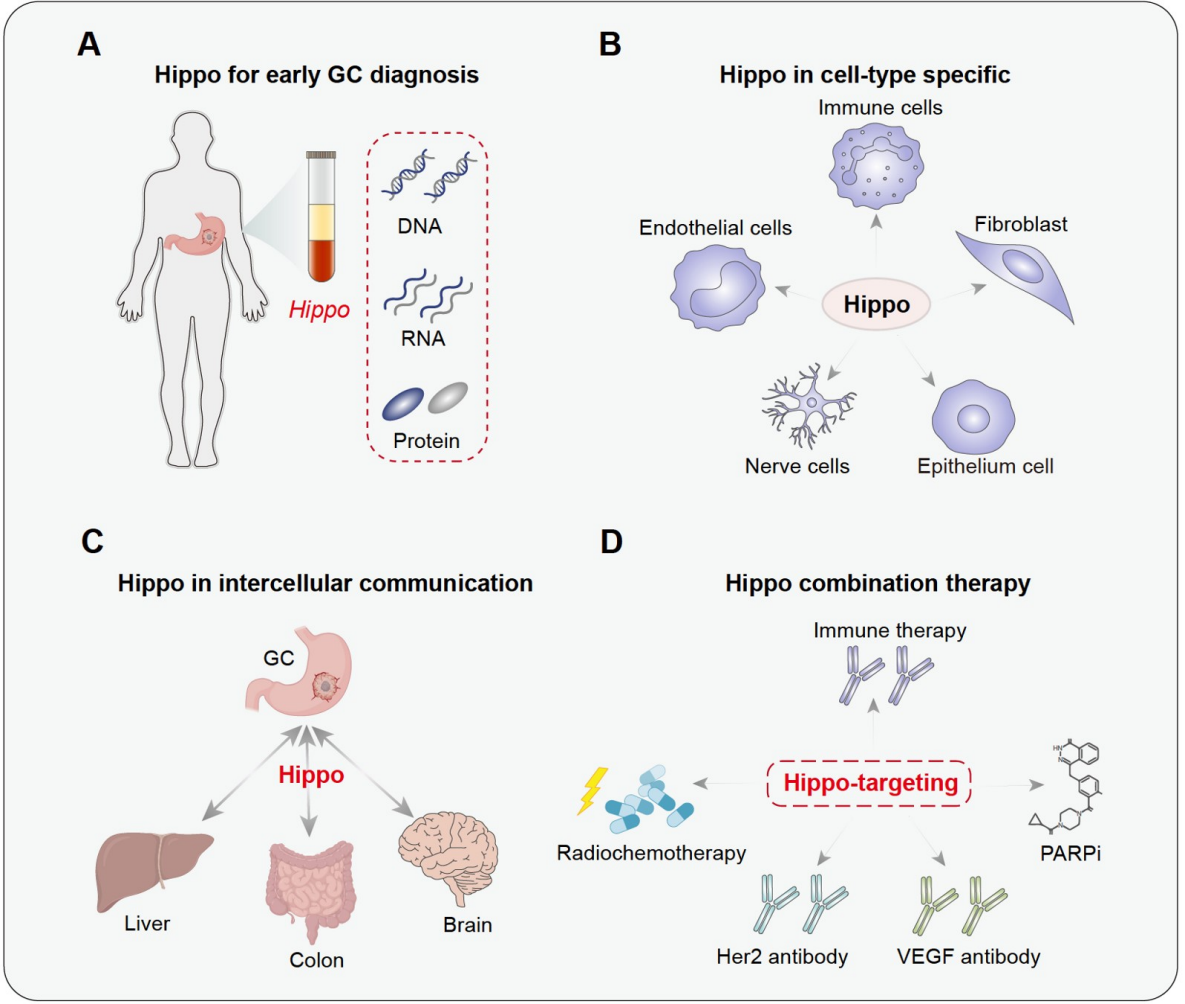
Figure 4 Future perspectives on Hippo signaling in GC (A) Identification of Hippo-related biomarkers for early GC detection using liquid biopsy-based techniques. (B) Cell-type specific regulatory roles of Hippo signaling in the GC microenvironment. (C) Potential roles of Hippo signaling in intercellular communication during GC distal metastasis. (D) Combination of Hippo-targeting strategy with current available GC therapies such as radio-chemotherapy, immune therapy, HER2 and VEGF antibodies, as well as PARP inhibitors for GC treatment.

First, although the activation and overexpression of YAP/TAZ are frequently observed in different types of tumors, there is still a lack of translational biomarkers related to the Hippo pathway for tumor diagnosis. As dysregulation of Hippo has been implicated in GC initiation, it is theoretically possible to identify Hippo-related biomarkers for early tumor diagnosis. In this regard, liquid biopsy-based techniques such as droplet digital PCR or next-generation sequencing have been extensively applied for early cancer diagnosis [ 126, 127] . Thus, further in-depth investigation is warranted to uncover clinical correlations or causal relationships of alterations in Hippo signaling or serological protein levels with GC occurrence, progression, and vulnerability to targeted therapy ( Figure 4A). In addition to early diagnosis, exactly how Hippo is dysregulated to dive GC initiation is also an interesting and important question awaiting dissection, especially in the context of specific disease or molecular subtype.

Second, the current understanding of Hippo in GC progression mainly focuses on characterizing how YAP is aberrantly activated and eventually leads to cell proliferation, invasion, and migration in tumor cells *in vitro*, which lacks TME properties such as dynamic cell‒cell interactions and cell-type specific regulatory contexts ( Figure 4B). For example, YAP was mostly found to be hyperactive in tumor cells, but its activity was recently revealed to be attenuated in GC-associated neutrophils for establishment of the immune-suppressive microenvironment, arguing for a cell-type specific [86]. In this regard, several scRNA-seq analyses have profiled the cellular ecosystem of both primary and metastatic GC [ 128– 130] , providing a good basis to delineate the roles of Hippo in a cell-type specific manner ( Figure 4B). To this end, cell-type-specific depletion of upstream kinases such as Lats1/2 or simple knock-in of a nuclear-activated form of YAP (such as the 5SA mutant) mouse models should be introduced to better explore the pathological modulation of Hippo in each cell type during GC development ( Figure 4B). Meanwhile, intercellular communications, either directly or indirectly, have been realized to play important roles in mediating tumor progression, especially distal metastasis. Whether and how Hippo signaling is involved in these processes also attracts researchers’ attention in fields including development and tumor biology ( Figure 4C). In this regard, state-of-the-art techniques such as synthetic notch receptors and intercellular proximity-dependent cell surface labelling strategies hold great promise to uncover Hippo-related cell‒cell communications and provide new insights into GC pathology or even treatment [ 131, 132] .

Finally, it should be noted that most Hippo-related therapeutic strategies are still in the preclinical stage with a few undergoing clinical trials, but none have been approved by the FDA or CFDA for clinical application [133]. On the one hand, as upstream kinases have additional roles beyond Hippo regulation, the “Hippo activation strategy” may trigger unpredictable side effects. Thus, the specific context of this targeting strategy must be thoroughly studied to avoid possible side effects. On the other hand, the issue of drug resistance should also be taken into consideration when developing these Hippo-targeting strategies. In this regard, exploring Hippo-based combination therapies such as pairing with radio-chemotherapy, immune checkpoint blockage, HER2 and VEGFR antibodies represents an attractive direction with great potential to overcome drug resistance ( Figure 4D).
